# Supplementary material for: Palladium and Platinum Complexes of the Antimetabolite Fludarabine with Vastly Enhanced Selectivity for Tumour over Non-Malignant Cells
Source: Molecules. 2023 Jul 2;28(13):5173. doi: 10.3390/molecules28135173 (PMC10343763; doi:10.3390/molecules28135173)
Supplement: Supplementary file 1 [file molecules-28-05173-s001.zip › molecules-2465131-supplementary.pdf]

- Supporting Information -

Palladium and platinum complexes of the antimetabolite fludarabine with vastly enhanced selectivity for tumour over non-malignant cells

Sebastian W. Schleser[1], Oleksandr Krytovych[1], Tim Ziegelmeier[1], Elisabeth Groß[2], Jana Kasparkova[3], Viktor Brabec[3], Thomas Weber[2], Rainer Schobert[1,*], and Thomas Müller[2]

[1] Organic Chemistry Laboratory, University Bayreuth, Universitaetsstrasse 30, 95447 Bayreuth, Germany
* Correspondence: Rainer.Schobert@uni-bayreuth.de

[2] University Clinic for Internal Medicine IV, Hematology/Oncology, Medical Faculty, Martin Luther University Halle-Wittenberg, Ernst-Grube-Str. 40, 06120 Halle (Saale), Germany

[3] Department of Biophysics, Faculty of Science, Palacky University, Slechtitelu 27, 783 71 Olomouc, Czech Republic

Conflicts of interest: There are no conflicts of interest

Source of funding: Deutsche Forschungsgemeinschaft grant Scho 402/12-2

Inhalt

NMR spectra of **2**, **3** and **4** 3

Stability studies of **3** and **4** 13

NMR spectra of **2**, **3** and **4**

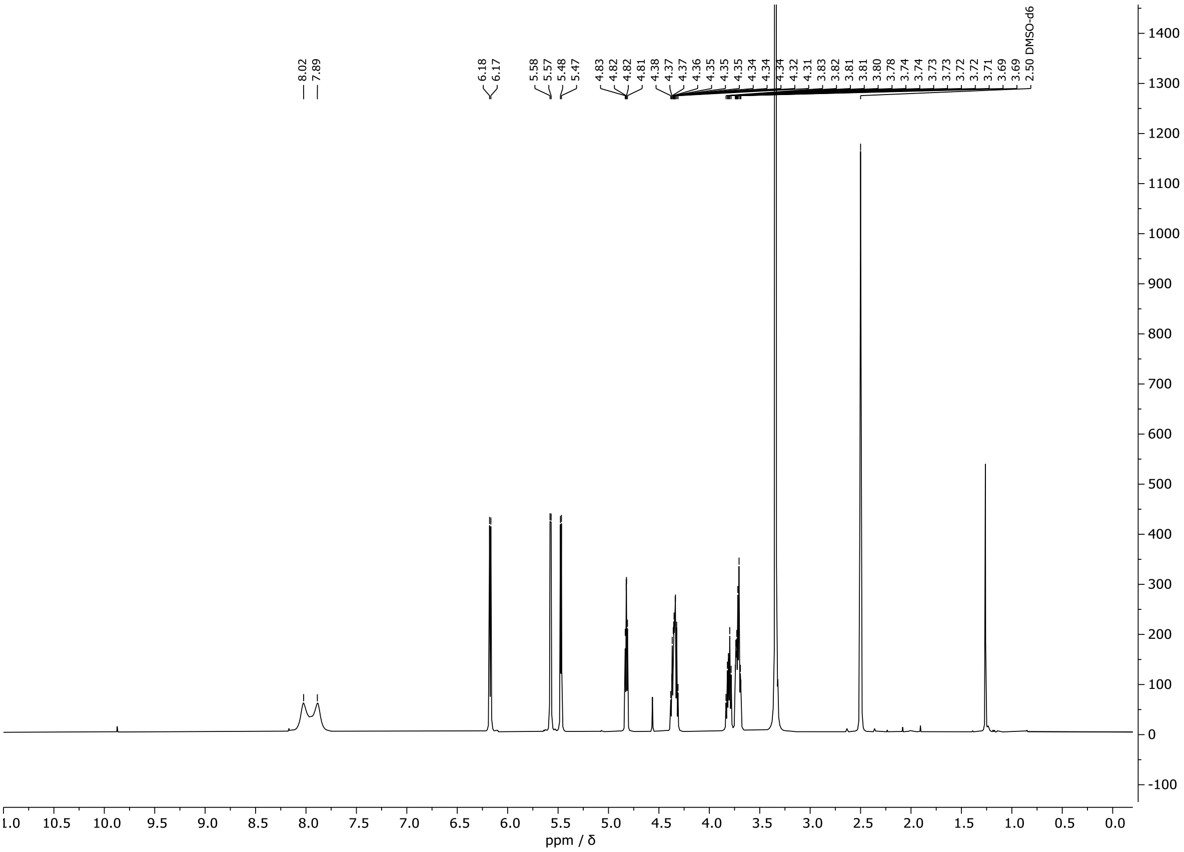

Figure S1. ^{1}H-NMR spectrum of **2** in DMSO-d_6.

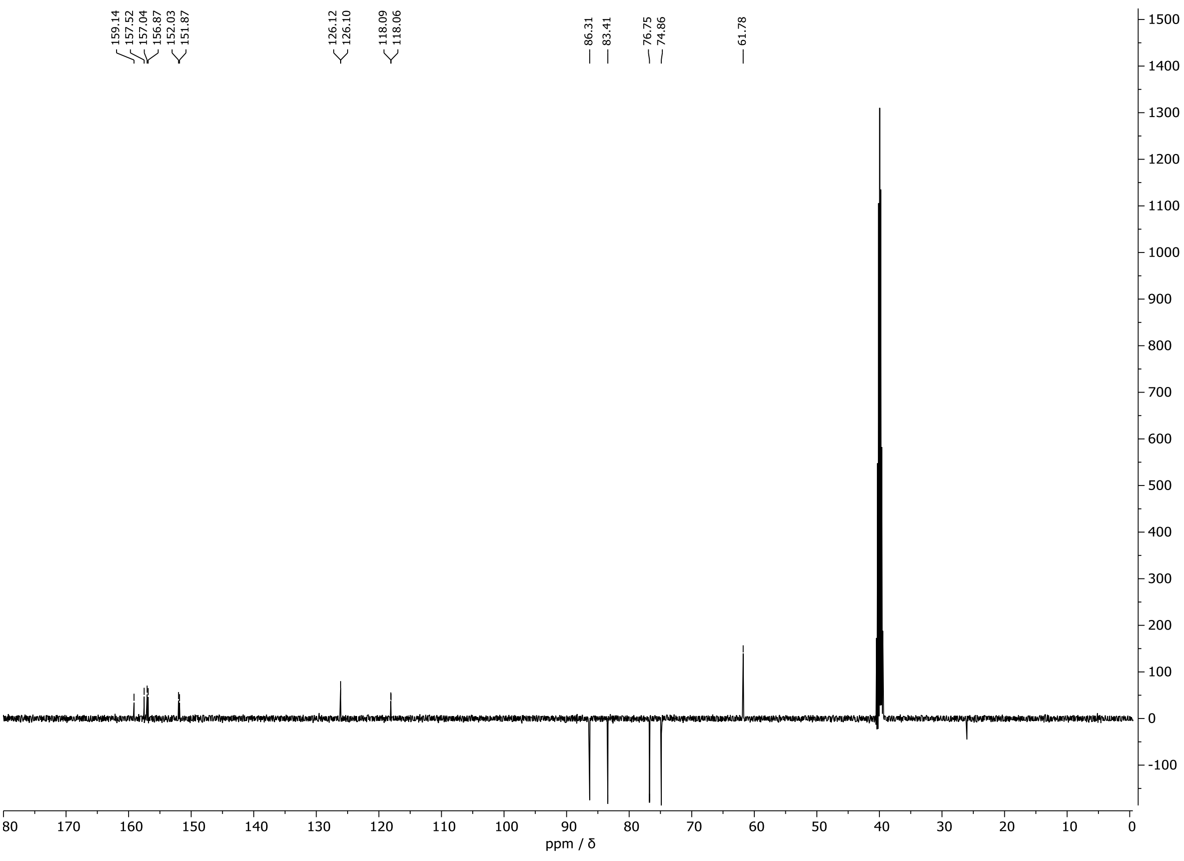

Figure S2. ^{13}C-NMR spectrum of **2** in DMSO-d$_6$.

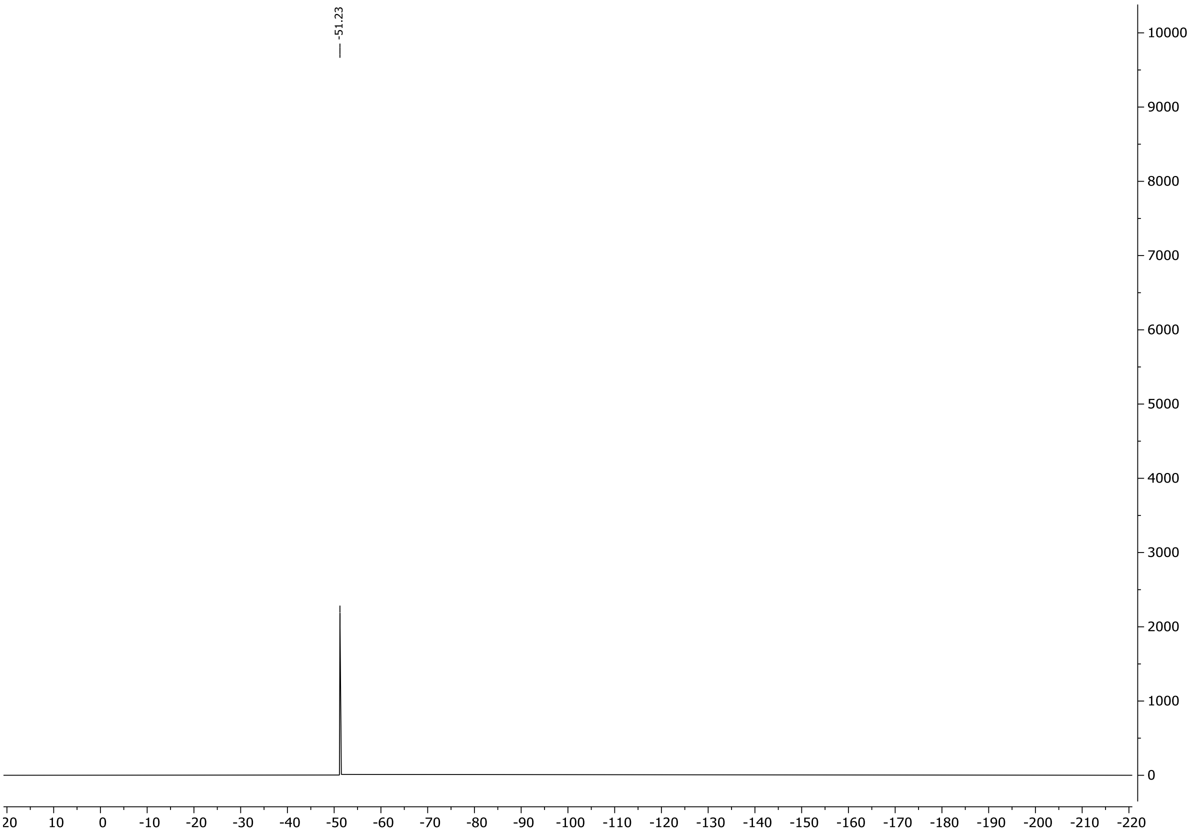

Figure S3. ^{19}F-NMR spectrum of **2** in DMSO-d$_6$.

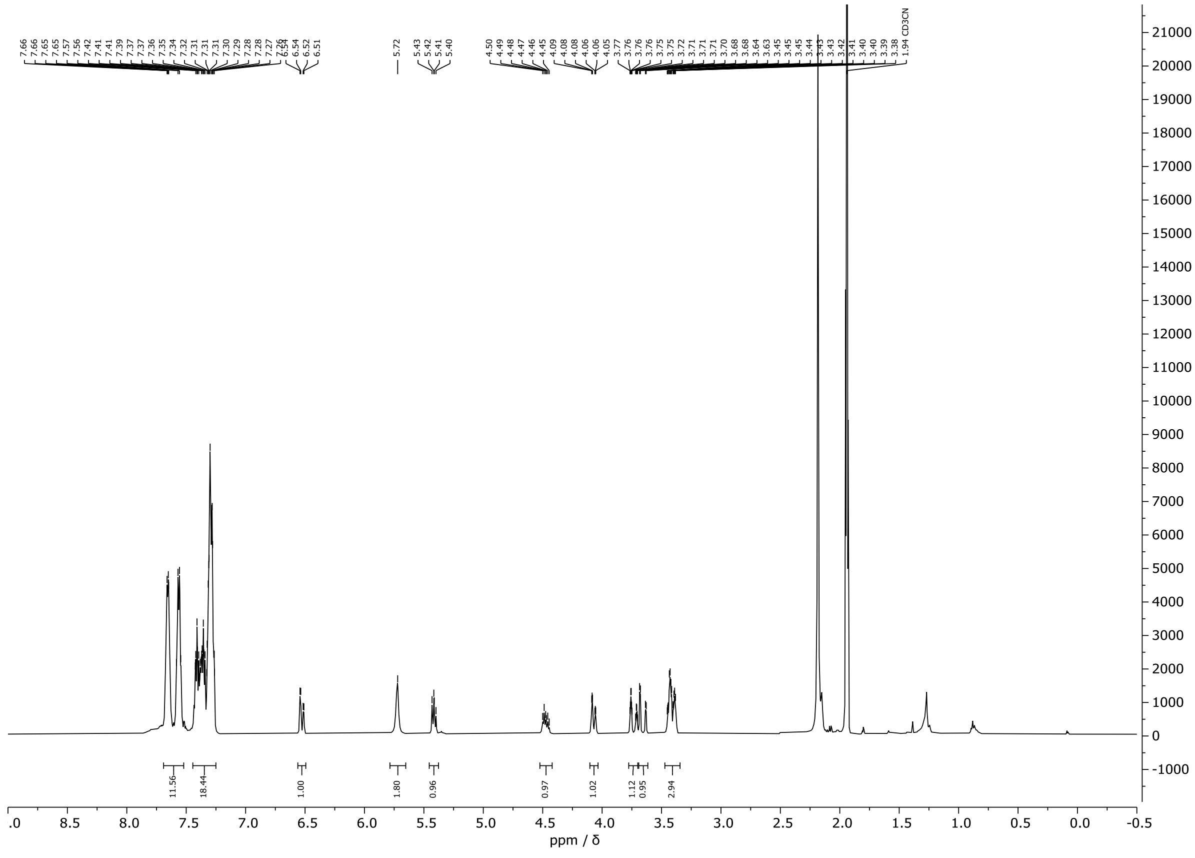

Figure S4. ^{1}H-NMR spectrum of **3a** in CD_3CN.

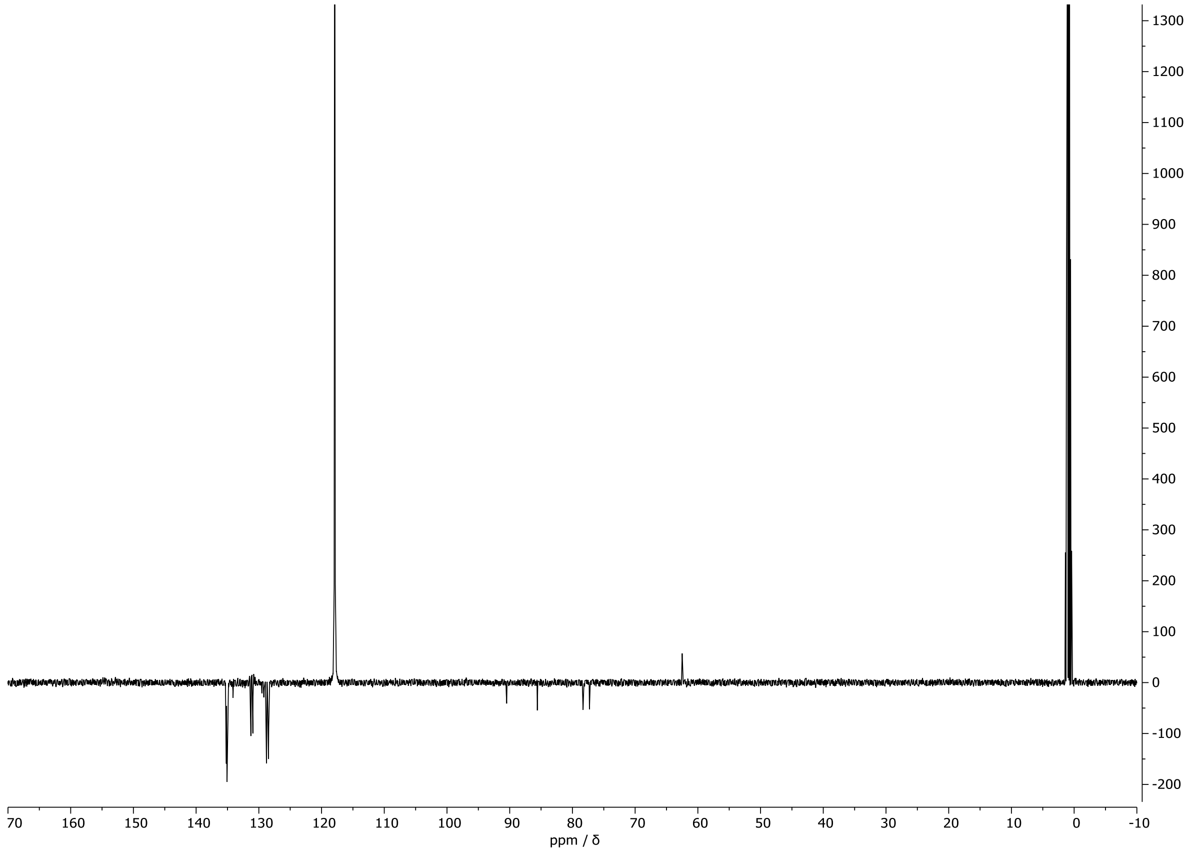

Figure S5. ^{13}C-NMR spectrum of **3a** in CD_3CN.

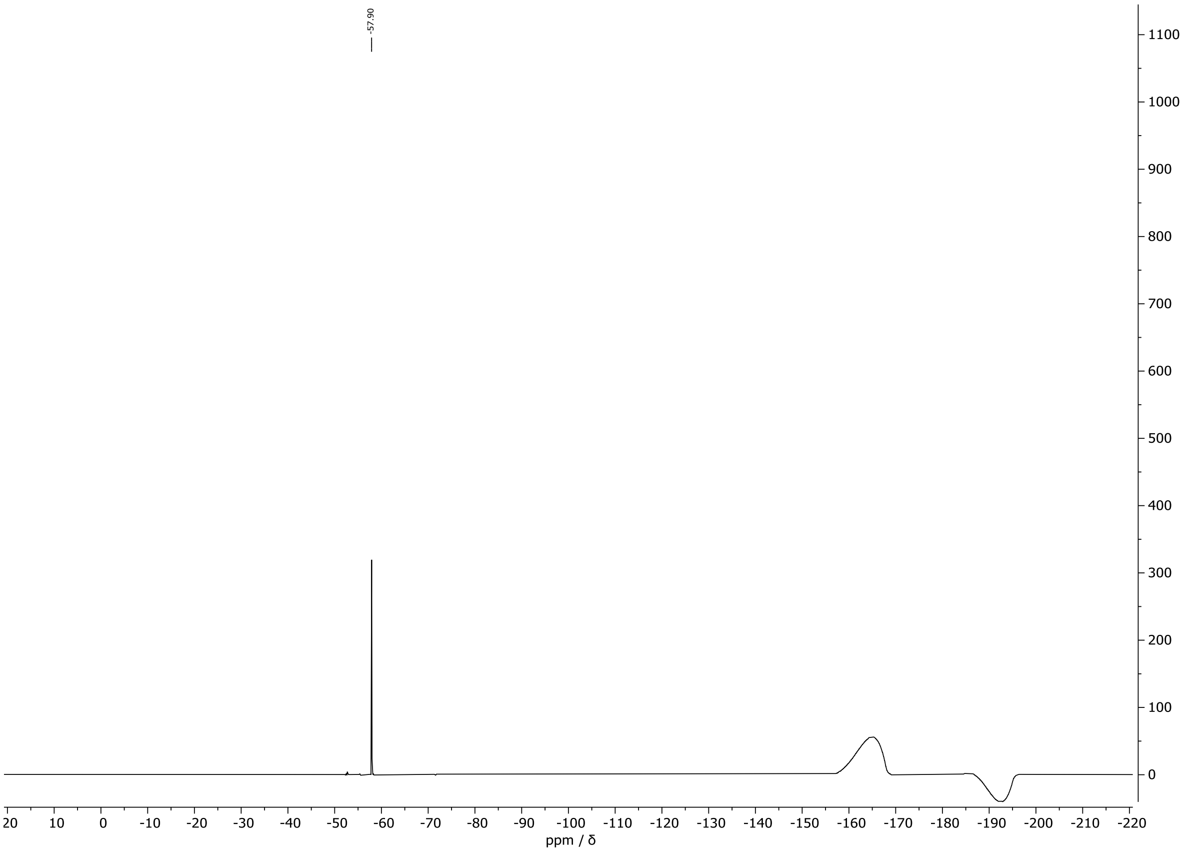

Figure S6. ^{19}F-NMR spectrum of **3a** in CD_3CN.

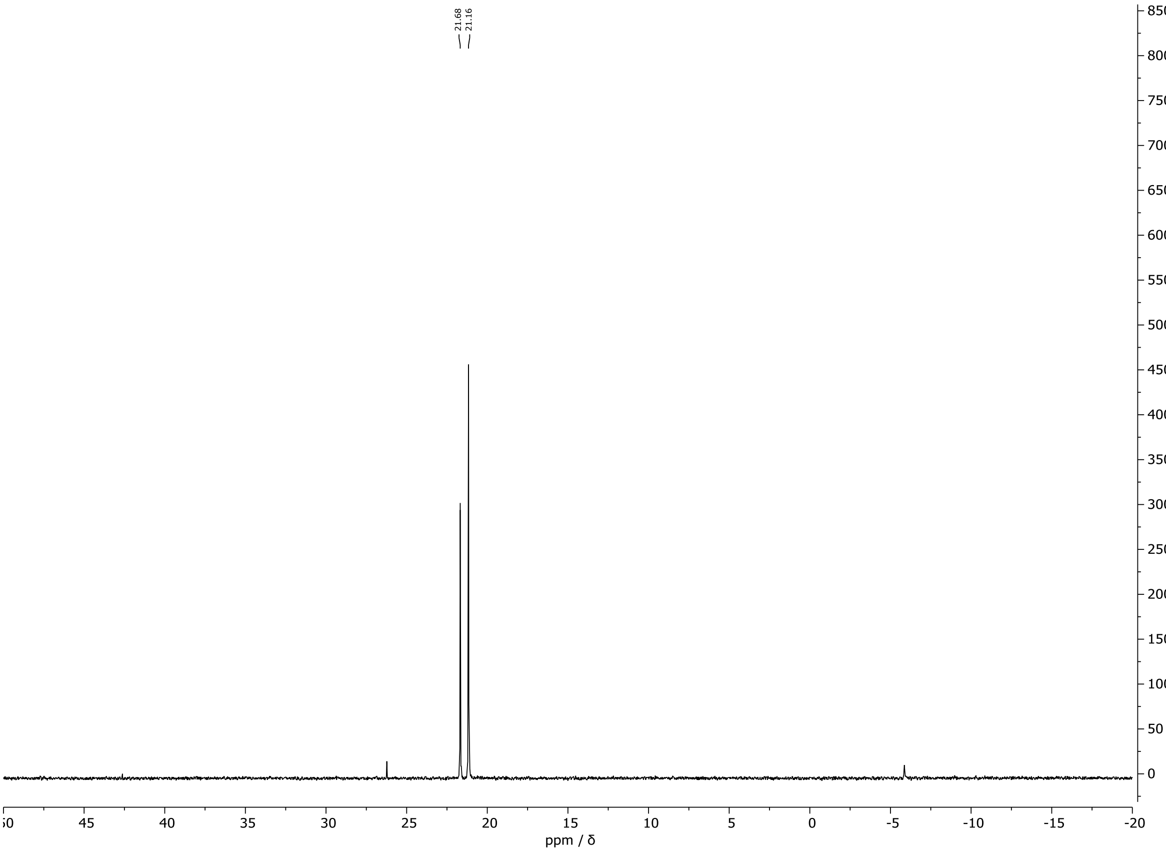

Figure S7. ^{31}P-NMR spectrum of **3a** in CD_3CN.

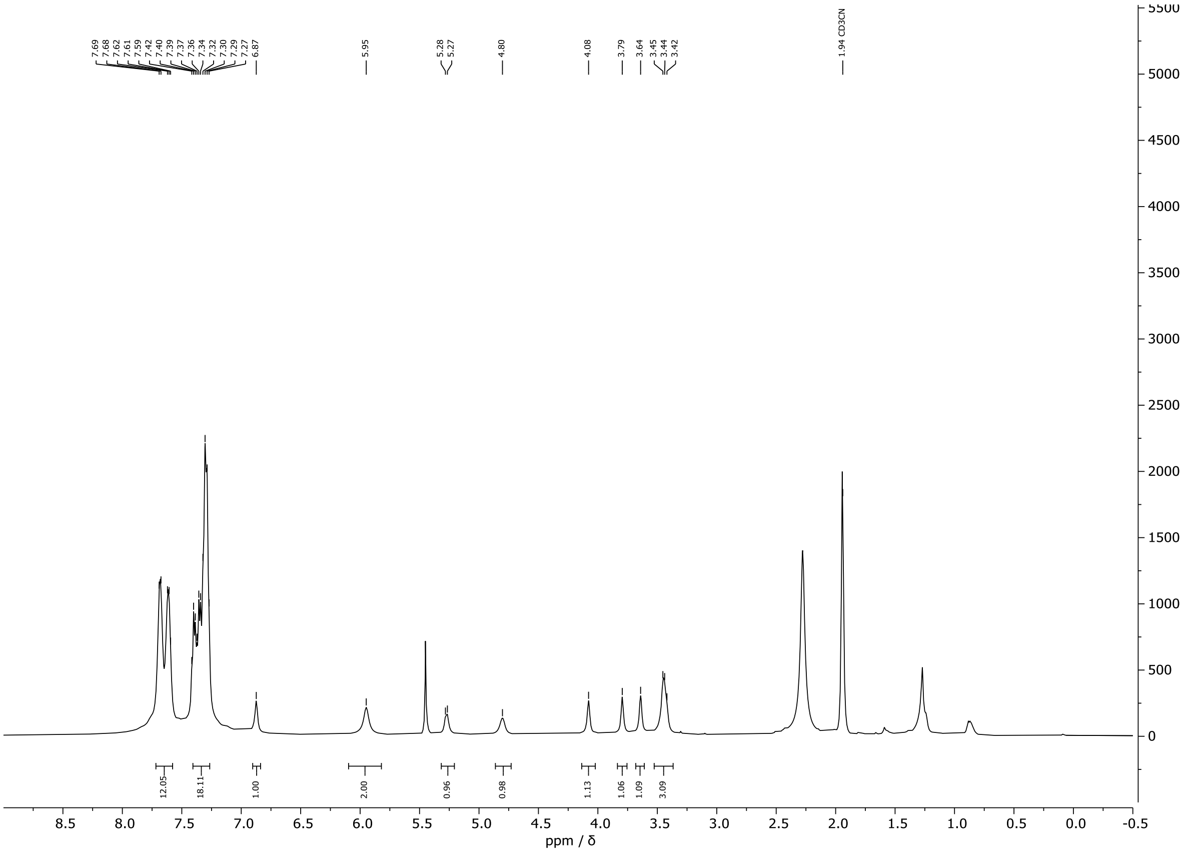

Figure S8. ^{1}H-NMR spectrum of **3b** in CD_3CN.

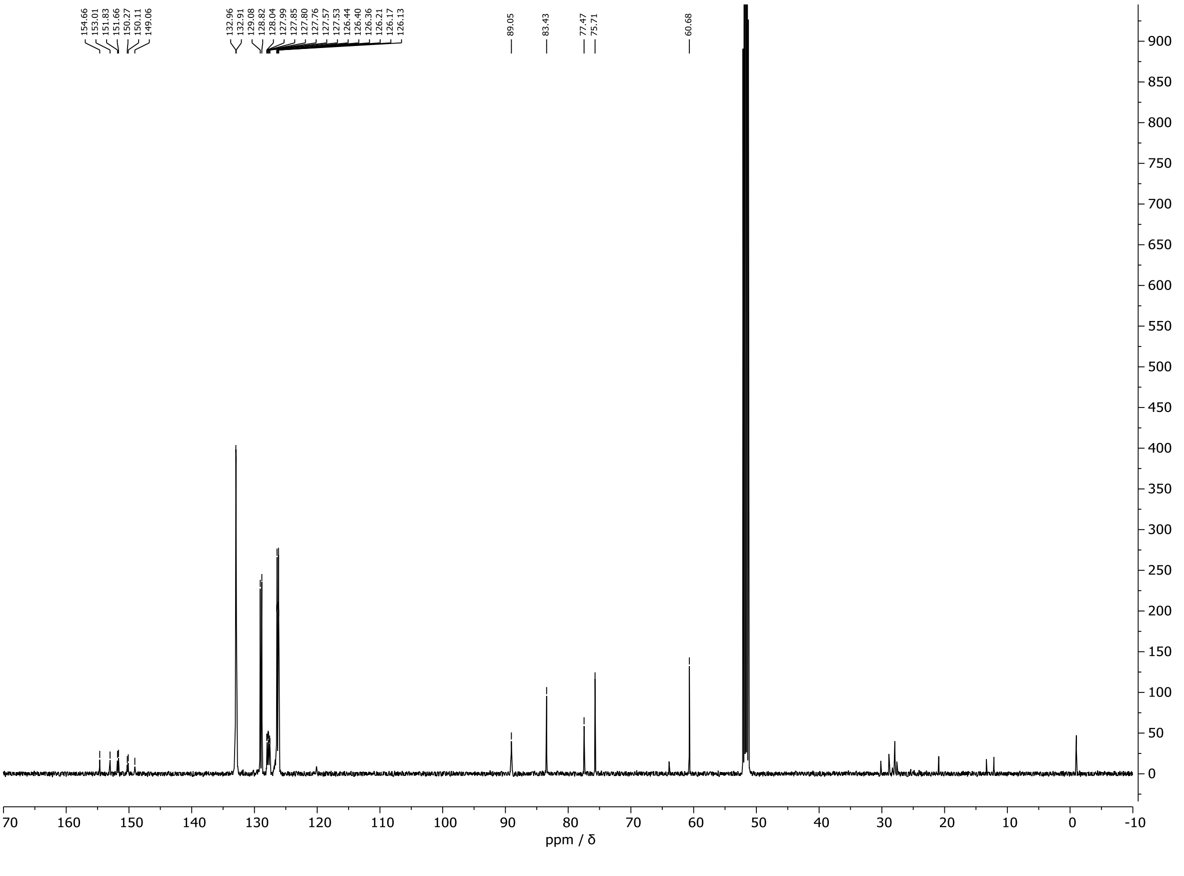

Figure S9. ^{13}C-NMR spectrum of **3b** in CD_2Cl_2.

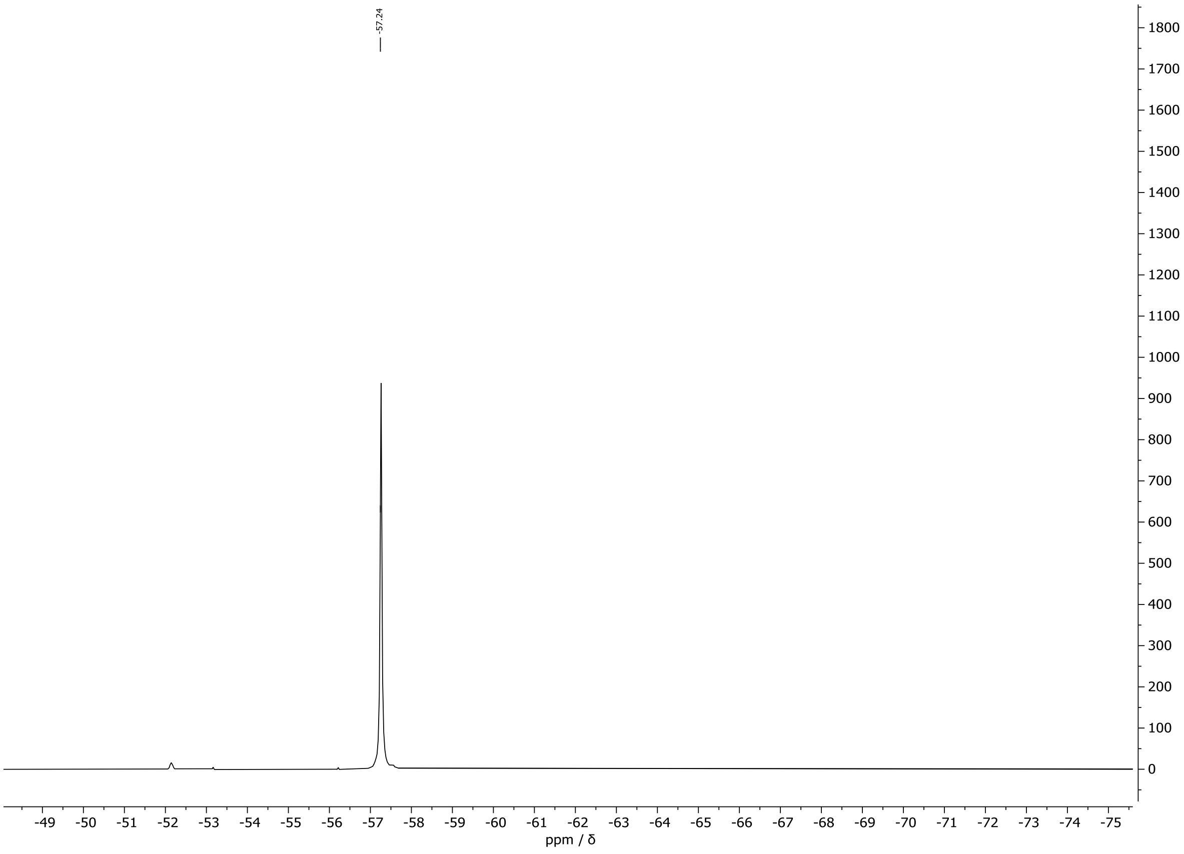

Figure S10. ^{19}F-NMR spectrum of **3b** in CD_2Cl_2.

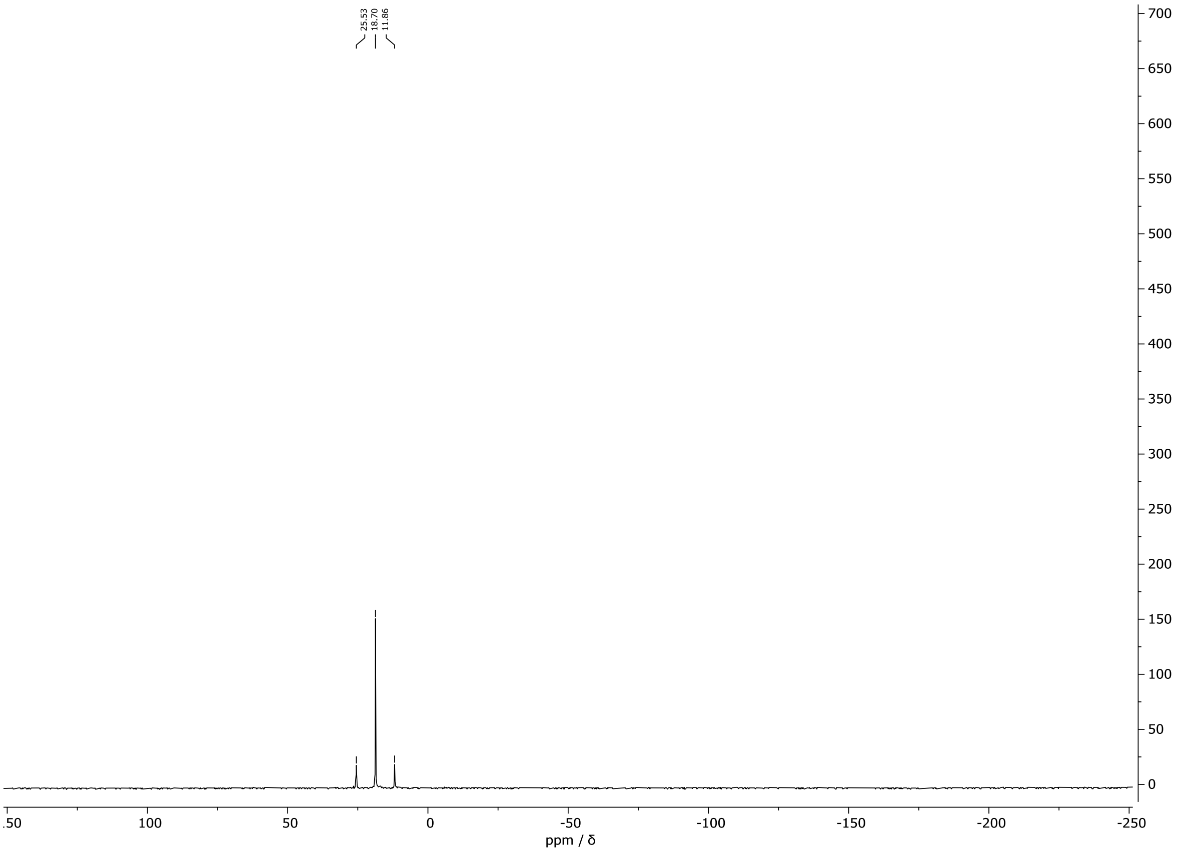

Figure S11. ^{31}P-NMR spectrum of **3b** in CD_2Cl_2.

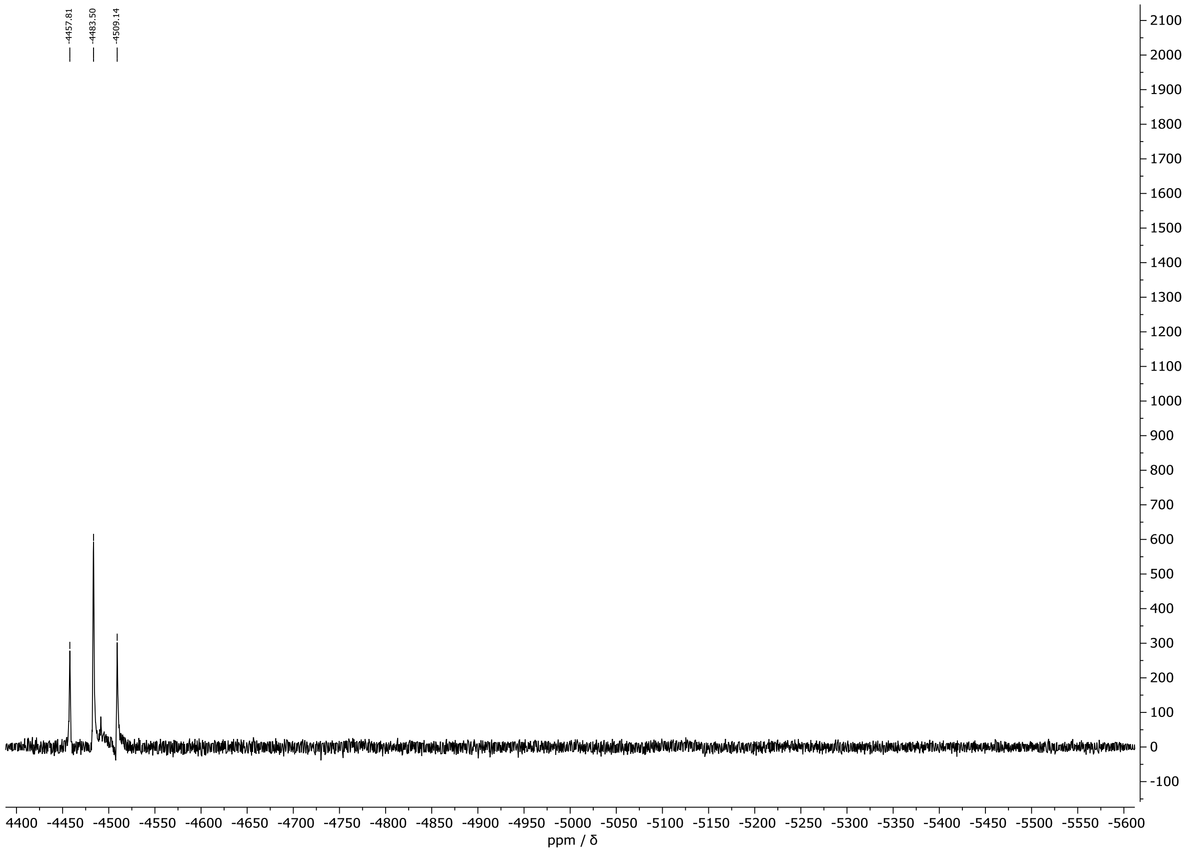

Figure S12. ^{195}Pt-NMR spectrum of **3b** in CD_2Cl_2.

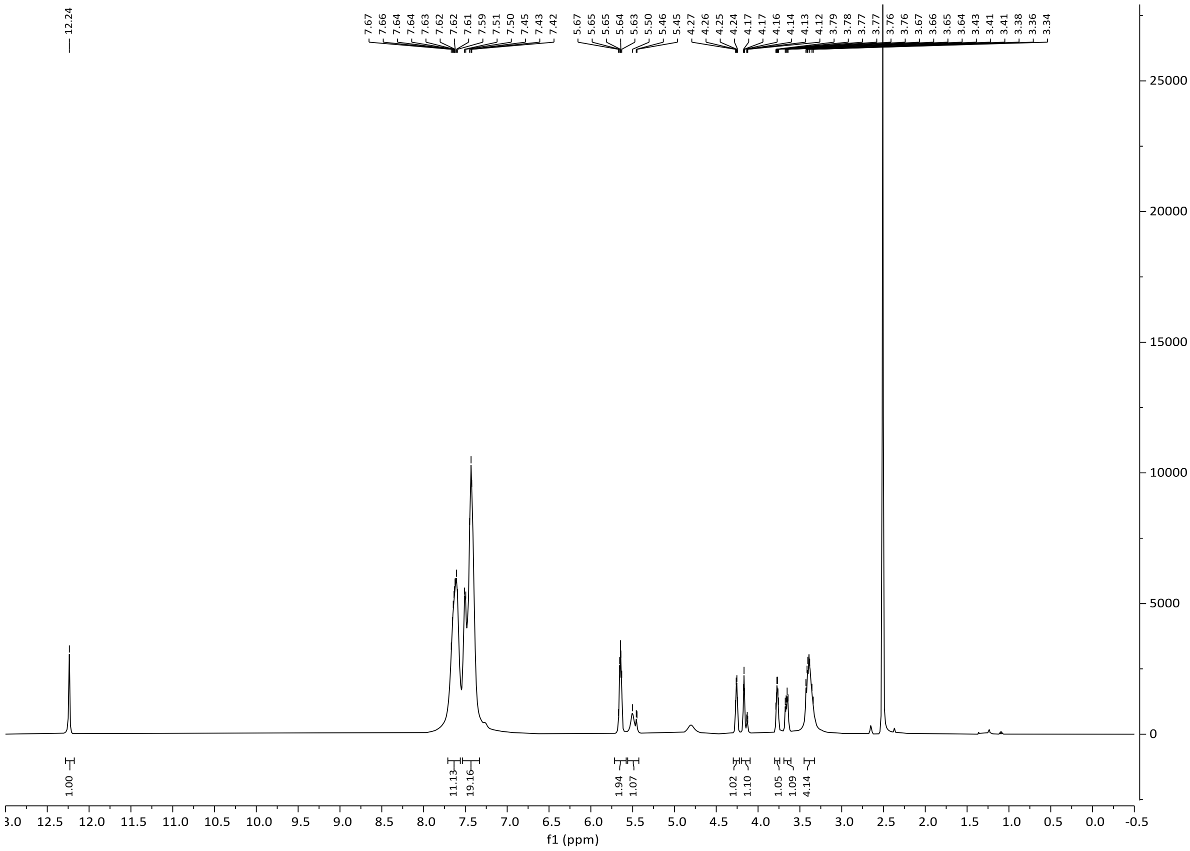

Figure S13. ^{1}H-NMR spectrum of **4a** in DMSO-d_6.

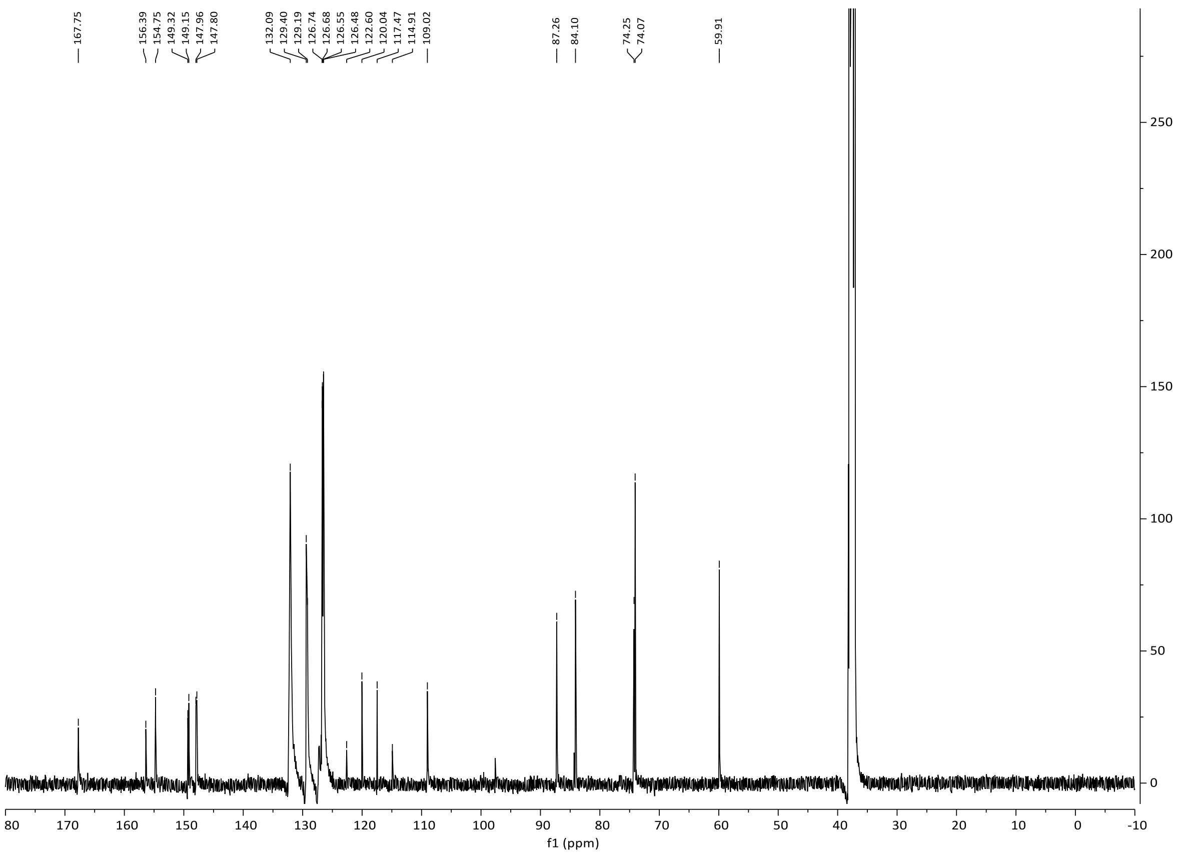

Figure S14. ^{13}C-NMR spectrum of **4a** in DMSO-d_6.

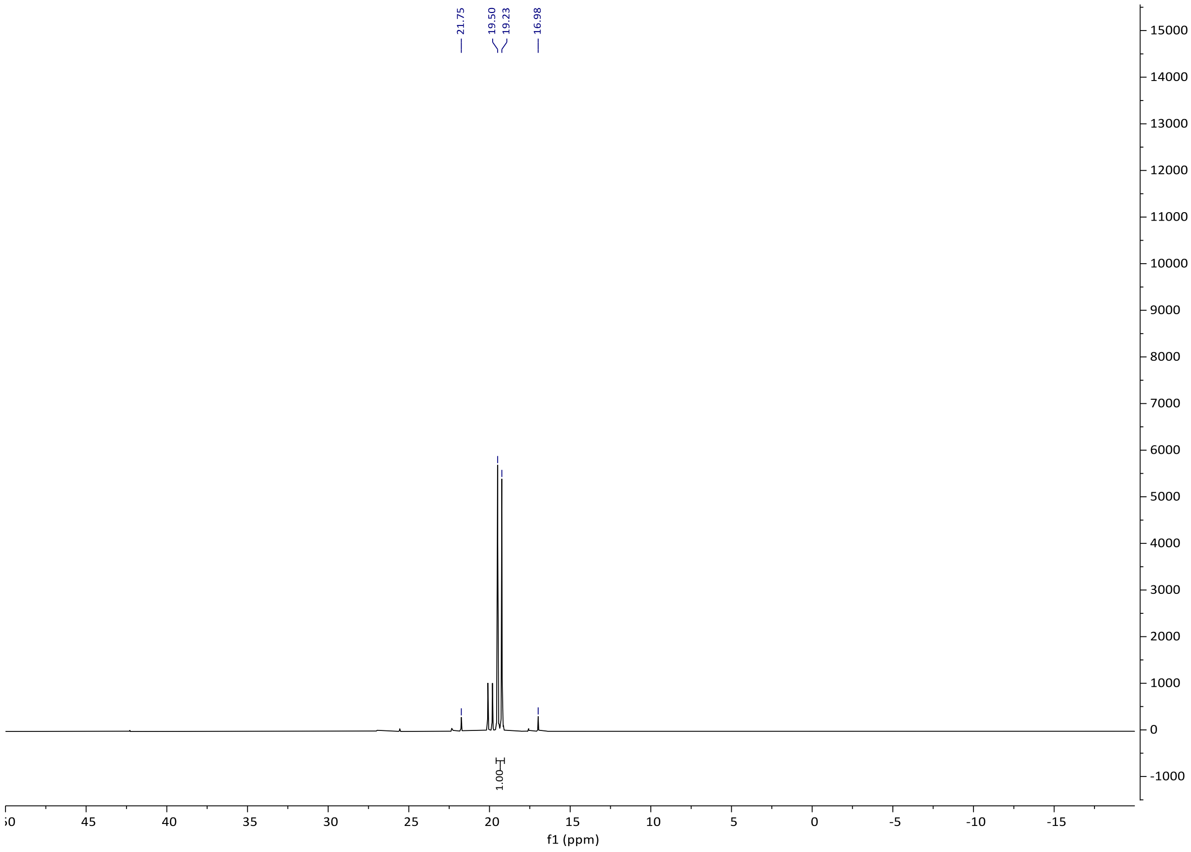

Figure S15. ^{31}P-NMR spectrum of **4a** in DMSO-d_6.

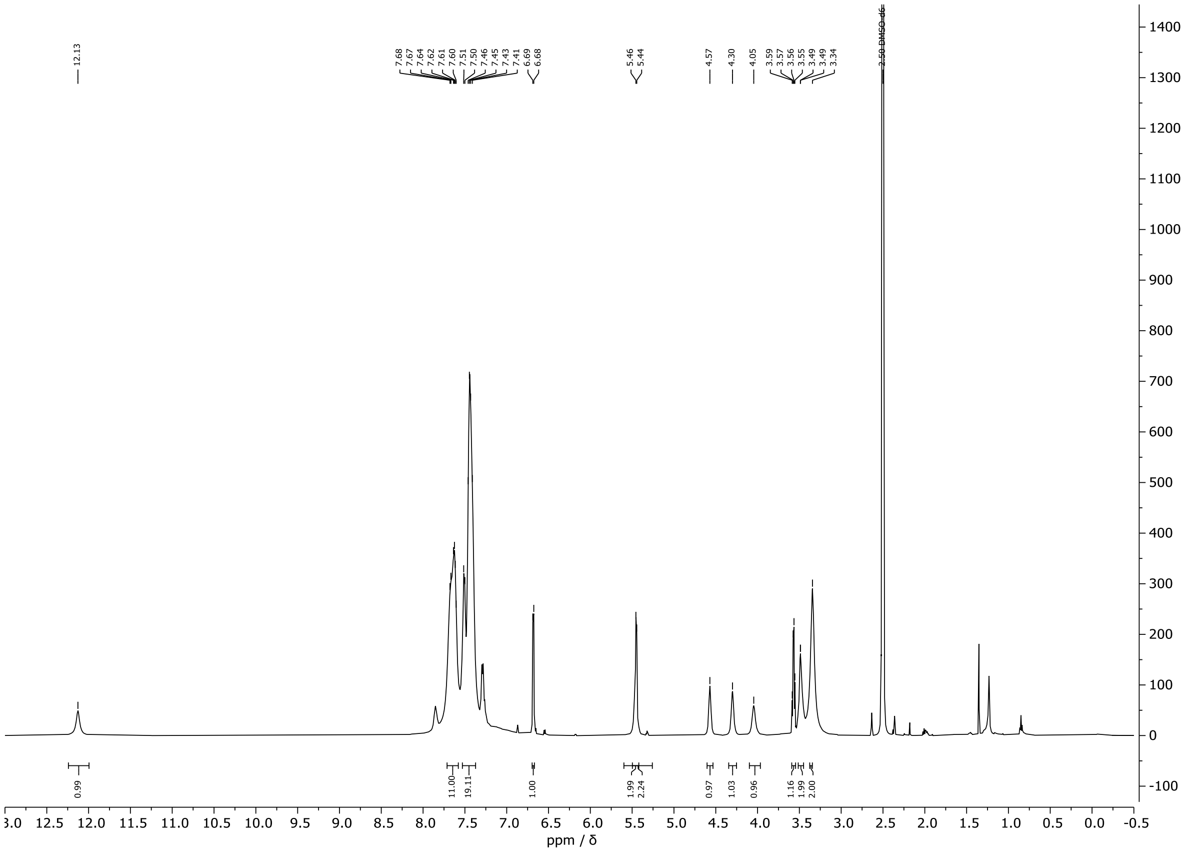

Figure S16. ^{1}H-NMR spectrum of **4b** in DMSO-d$_6$.

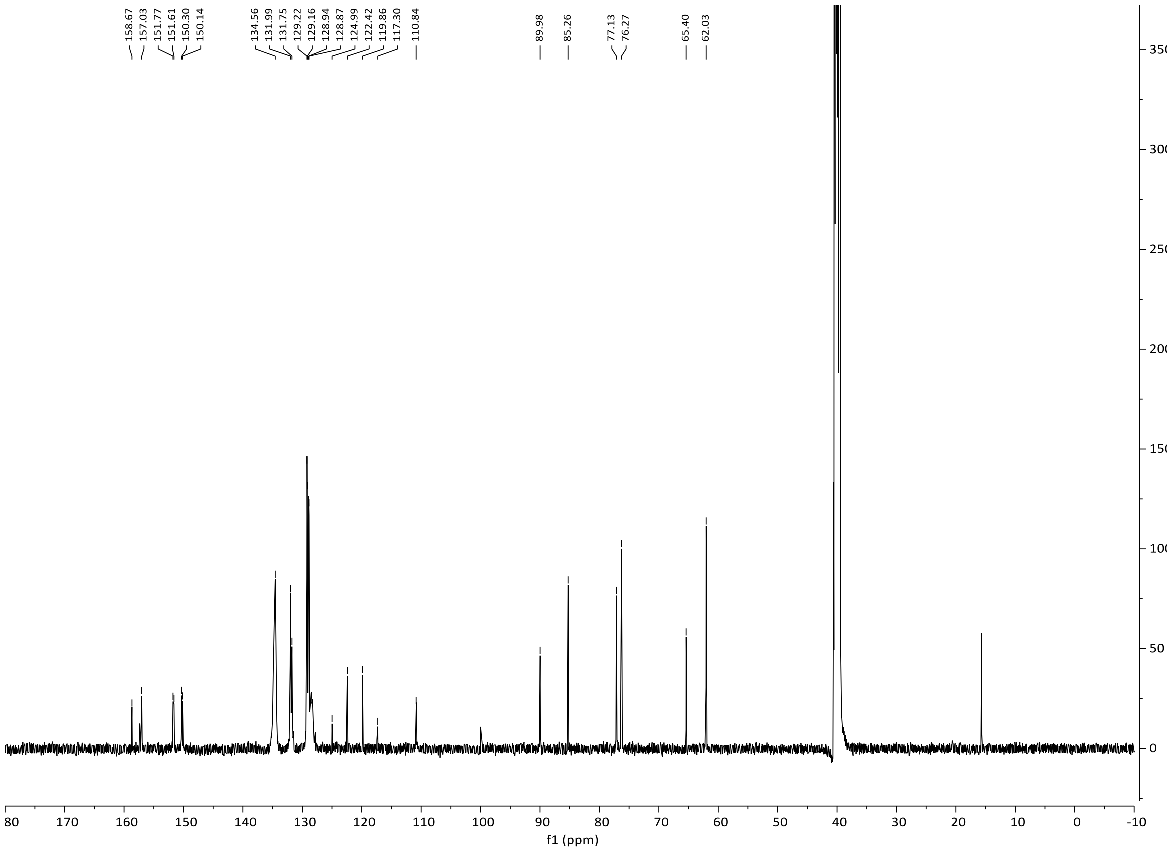

Figure S17. ^{13}C-NMR spectrum of **4b** in DMSO-d$_6$.

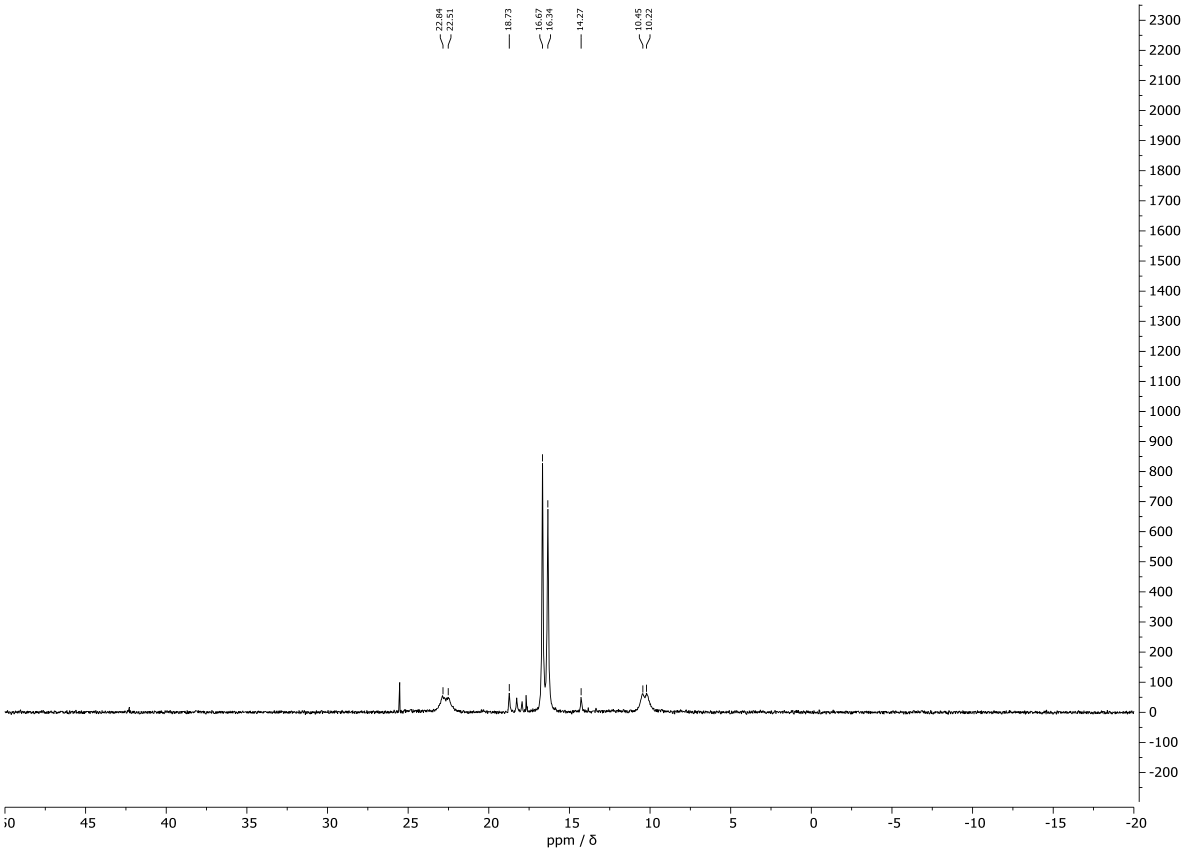

Figure S18. ^{31}P-NMR spectrum of **4b** in DMSO-d$_6$.

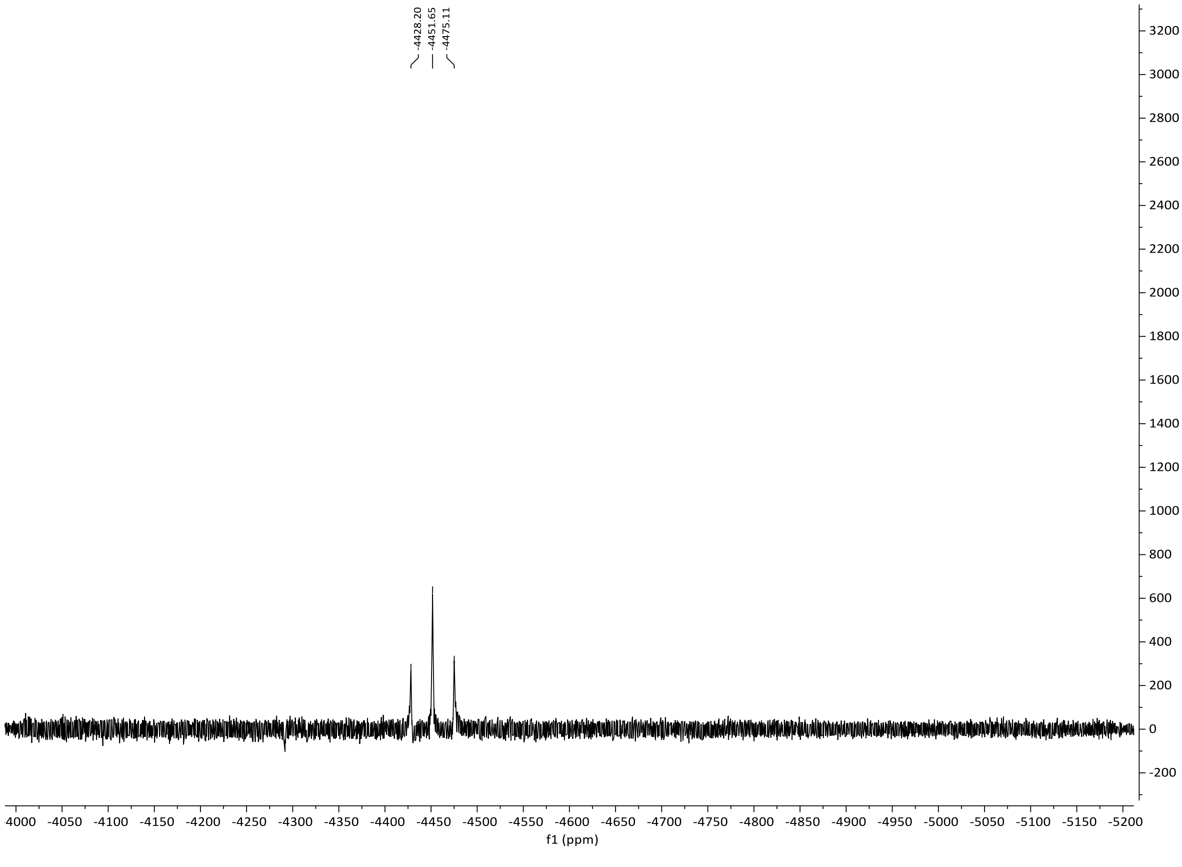

Figure S19. ^{195}Pt-NMR spectrum of **4b** in DMSO-d$_6$.

Stability studies of **3** and **4**

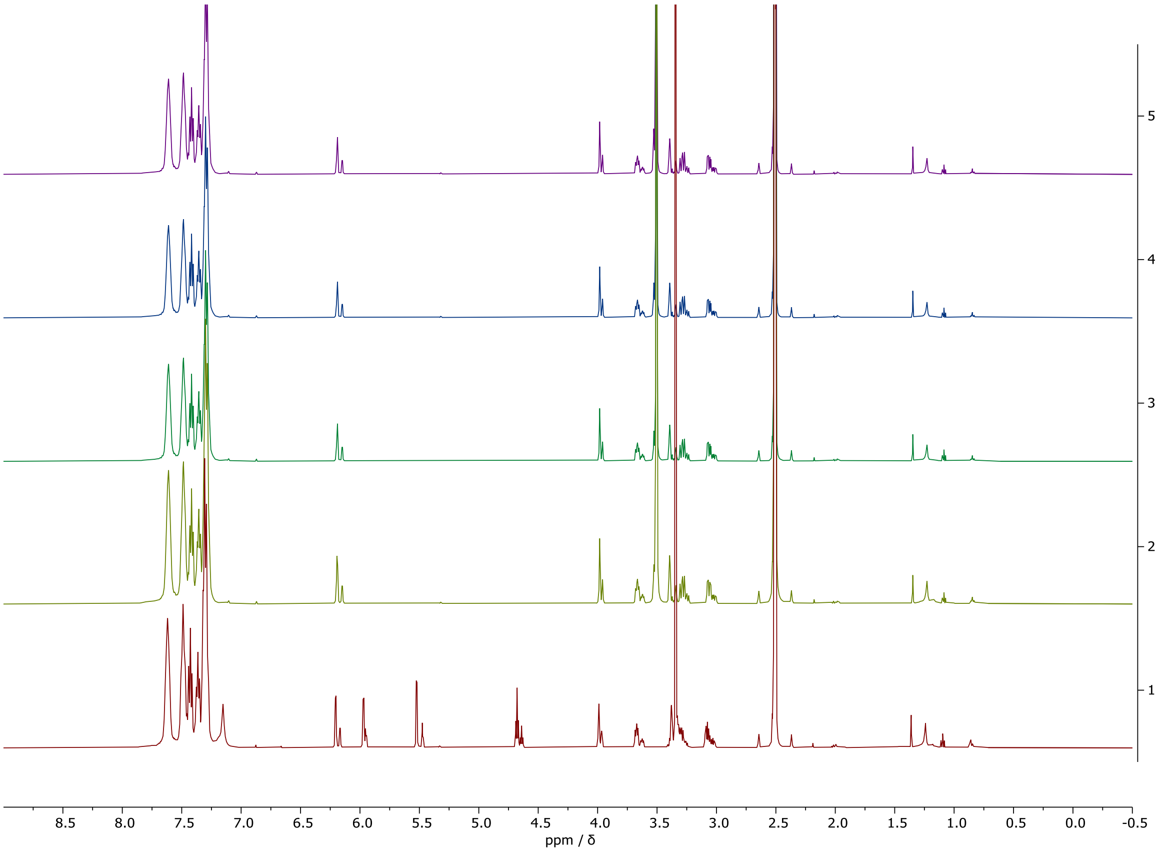

Figure S20. ^{1}H-NMR spectrum of **3a** in DMSO-d_6 (red), + 5% D_2O after 0h (light green), 24h (dark green), 48h (blue) and 72h (purple).

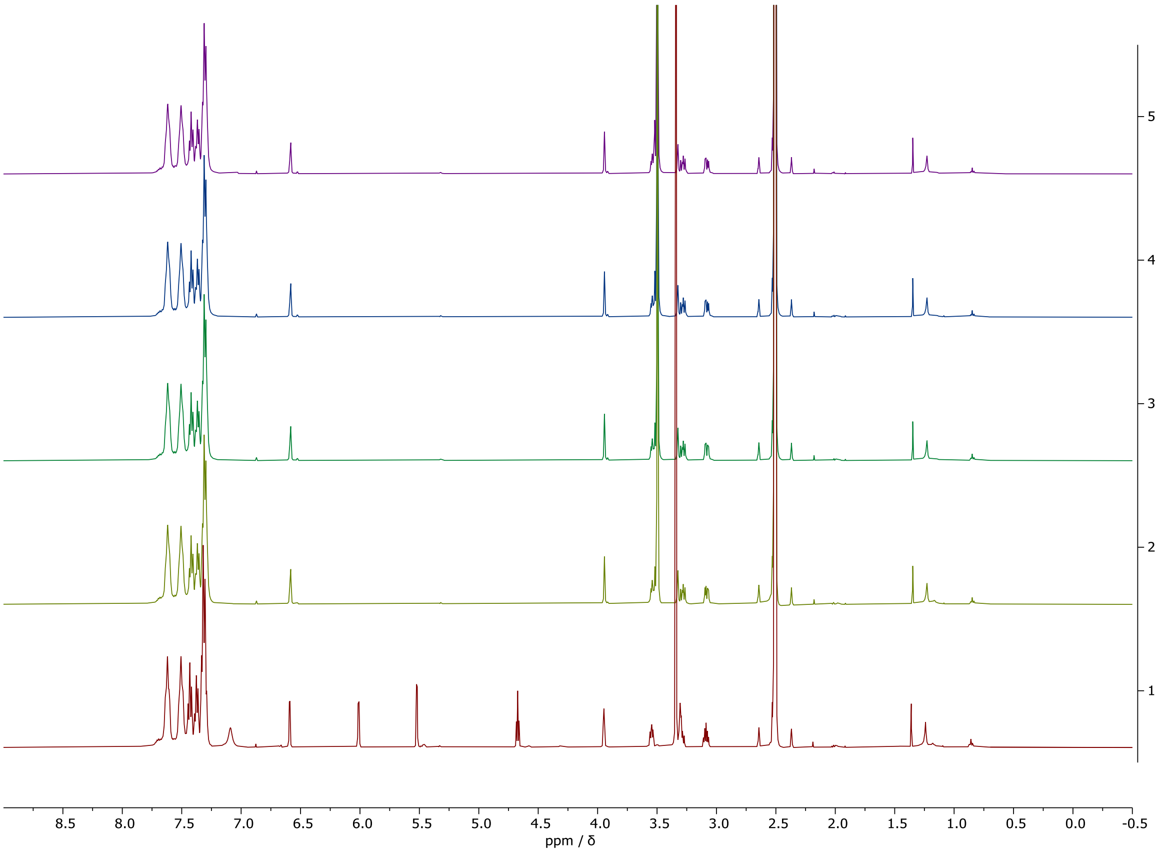

Figure S21. ^{1}H-NMR spectrum of **3b** in DMSO-d$_6$ (red), + 5% D_2O after 0h (light green), 24h (dark green), 48h (blue) and 72h (purple).

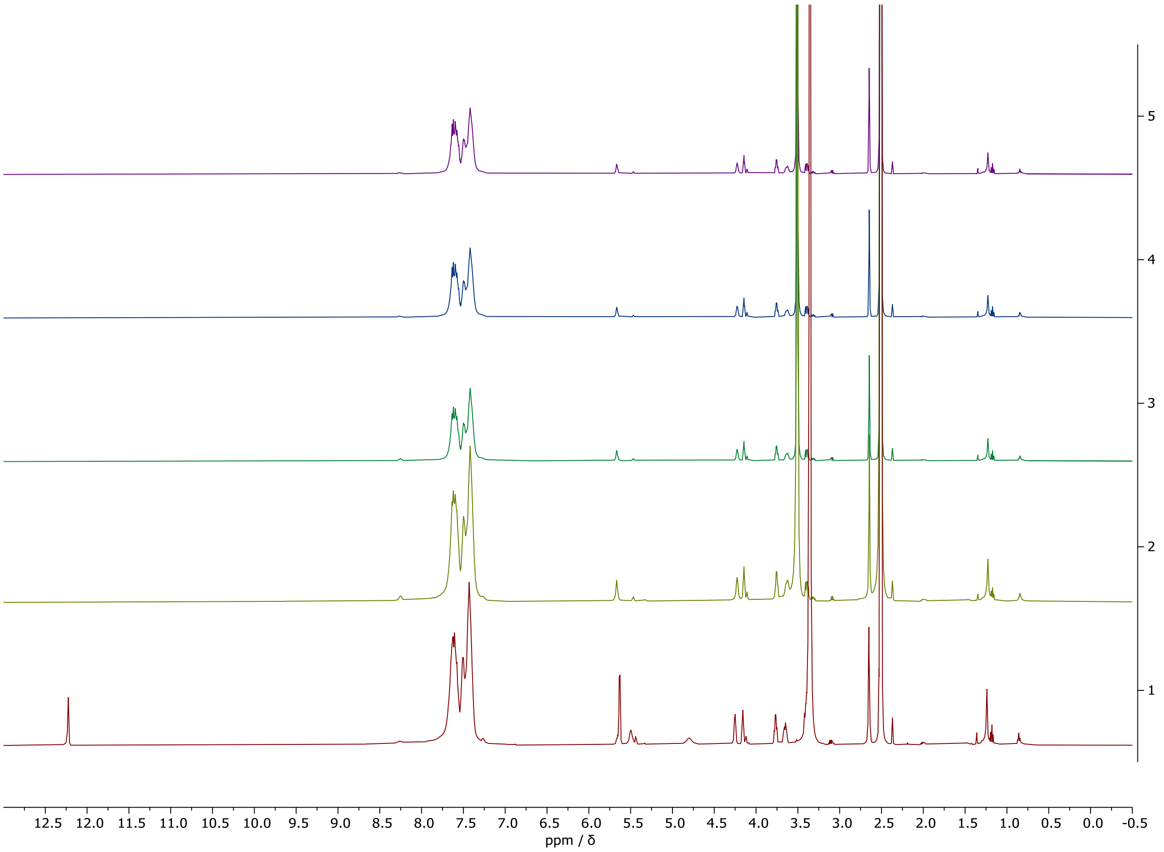

Figure S22. ^{1}H-NMR spectrum of **4a** in DMSO-d$_6$ (red), + 5% D_2O after 0h (light green), 24h (dark green), 48h (blue) and 72h (purple).

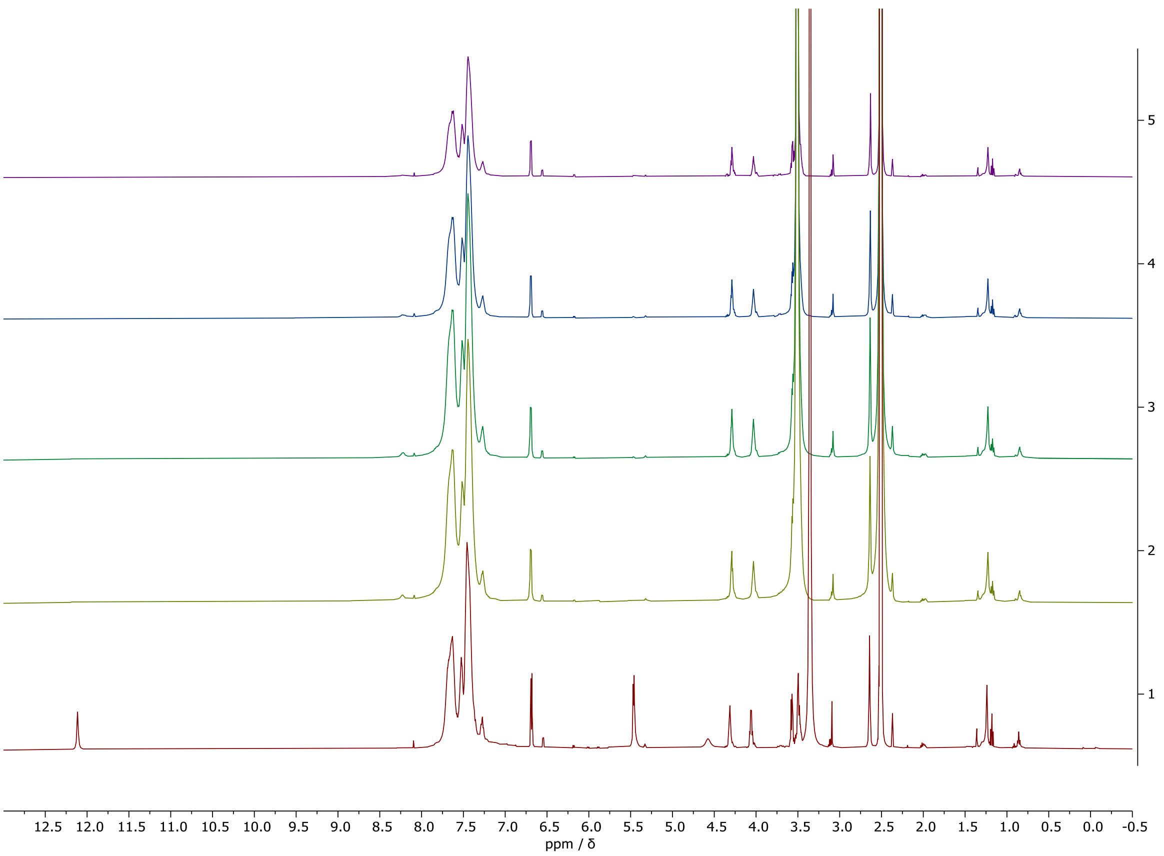

Figure S23. ^{1}H-NMR spectrum of **4b** in DMSO-d_6 (red), + 5% D_2O after 0h (light green), 24h (dark green), 48h (blue) and 72h (purple).
